# Application of Closed Incision Negative Pressure Wound Therapy in Ventral Hernia Repair Surgery Using a Polypropylene Mesh: A Randomized Clinical Trial

**DOI:** 10.3390/medicina60091548

**Published:** 2024-09-22

**Authors:** Petr Jelinek, Jan Hrubovcak, Radovan Hajovsky, Jan Velicka, Martin Pies

**Affiliations:** 1 Department of Surgery, University Hospital Ostrava, 17. listopadu 1790/5, 708 00 Ostrava-Poruba, Czech Republic; jan.hrubovcak@fno.cz; 2 Department of Surgical Studies, Faculty of Medicine, University of Ostrava, Syllabova 19, 703 00 Ostrava-Vítkovice, Czech Republic; 3 Department of Cybernetics and Biomedical Engineering, Faculty of Electrical Engineering and Computer Science, VSB-Technical University of Ostrava, 17. listopadu 2172/15, 708 00 Ostrava-Poruba, Czech Republic; radovan.hajovsky@vsb.cz (R.H.); jan.velicka@vsb.cz (J.V.)

**Keywords:** negative pressure therapy, polypropylene mesh, randomized trial, wound infection, aseptic wounds, ventral hernia repair

## Abstract

*Background and Objectives*: Surgical site infections (SSIs) are a significant complication following ventral hernia repair, potentially leading to prolonged hospital stays and increased morbidity. This study aimed to evaluate whether closed incision negative pressure wound therapy (ciNPWT) reduces the incidence of SSI after ventral hernia repair with polypropylene mesh compared to standard wound care. *Materials and Methods*: A randomized study was conducted with 100 patients undergoing ventral hernia repair using a polypropylene mesh. Participants were divided into two groups: a control group (n=50), which received standard sterile gauze dressing with an iodine-based disinfectant, and an intervention group (n=50), treated with the ciNPWT system (Vivano^®^ by HARTMANN) for 5 days postoperatively. The primary outcome was the incidence of SSI within one year after surgery. Secondary outcomes included the influence of factors such as age, sex, smoking status, and hernia size on SSI occurrence. The study was approved by the Ethics Committee at the University Hospital Ostrava, adhering to the ethical standards of the Helsinki Declaration. *Results*: The incidence of SSI was lower in the ciNPWT group compared to the standard care group (4% vs. 12%), though this difference did not reach statistical significance. No significant effect of sex or smoking status on SSI was observed. The control group had a shorter mean length of hospital stay. Larger hernias in the non-ciNPWT group were more prone to SSIs, as expected. *Conclusions*: Although limited by a small sample size, the findings suggest that ciNPWT may be associated with a reduced rate of SSI following ventral hernia repair. Further studies with larger populations are needed to confirm these results.

## 1. Introduction

Negative pressure wound therapy (NPWT) has been in use for over 30 years. Originally, its application was limited to treating exuding wounds or open wounds that could not be closed primarily, such as fistulae [1]. However, its use has expanded significantly, and it is now successfully applied to even sterile surgical wounds with primary closure, aiming to further reduce early wound complications such as seromas and surgical site infections (SSIs). This approach is known as ciNPWT (closed incision negative pressure wound therapy), where NPWT is applied to a clean and sutured incision immediately after surgery.

The effectiveness of ciNPWT is based on several mechanisms. Continuous negative pressure applied to a sutured wound helps stabilize it mechanically, promoting healing. Additionally, it enhances wound drainage through surface suction and reduces fluid accumulation in the wound by sealing off lymphatic vessels and capillaries. The pressure gradient also helps prevent bacterial translocation from the skin’s microbial flora into the surgical incision. The positive impact of ciNPWT on wound healing after elective procedures has been supported by various works, including those conducted by Berner-Hansen [2], Gombert [3], Scalise [4] and Hrubovcak [5].

## 2. Materials and Methods

### 2.1. Data Source

**Figure 1:**
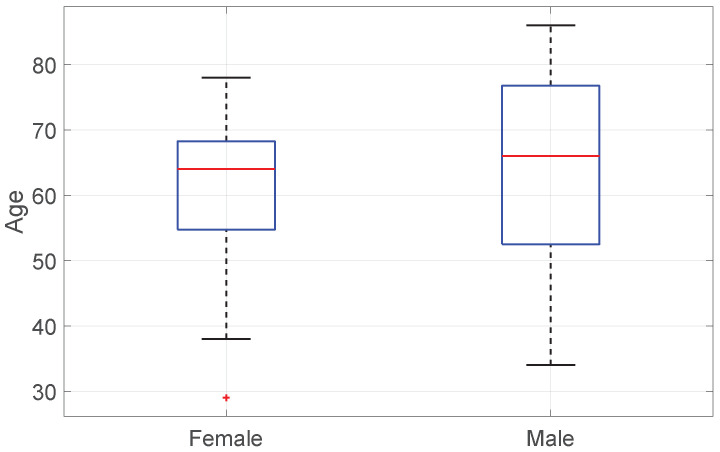
Boxplot of age by sex. On each blue box, the central red mark indicates the median, and the bottom and top edges of the box indicate the 25th and 75th percentiles, respectively. The whiskers extend to the most extreme data points not considered outliers, and the outliers are plotted individually using the + marker red symbol.

We evaluated 100 patients (Female 49, Male 51) undergoing ventral hernia repair through open surgery with a polypropylene mesh at a single hospital. The study was approved by the Ethics Committee at the University Hospital Ostrava, in accordance with the ethical standards of the Helsinki Declaration of 1975, as amended in 2013 (reference number 75/2023). The study had a retrospective, observational, and open-label design (ClinicalTrials. gov registration: NCT02893891). A retrospective observational study of patients following surgery procedures was carried out over the course of two years (2021–2023) at the Department of Surgery of the University Hospital Ostrava. From all the patients who underwent surgery at our center, we selected patients who met the entry criteria and were also willing to participate in a more time-consuming project. The age distribution of the patient cohort is illustrated in Figure 1. These patients were randomized by an envelope method into two groups. The patients were assigned a number. Those with an even number were placed in the first group and the rest with an odd number in the second group. The assignment process was carried out by a member of the Department of Surgery who was not involved in the patients’ care. The first group, consisting of 50 patients, had their surgical wounds treated with standard sterile gauze and an iodine disinfectant. The second group, also comprising 50 patients, received ciNPWT immediately after skin closure, with the therapy applied for a duration of 5 days. This was based on a study [6] that reports the use of ciNPWT for a duration of at least 7 days after surgery. The whole week was, however, deemed unnecessarily long, and a shorter period was chosen instead. Currently, the authors are investigating whether an even shorter period, such as 3–4 days, might be sufficient. We used a commercially available NPWT system, Vivano^®^ by HARTMANN, with the pressure set to −90 mmHg (−120 hPa).

**Figure 2:**
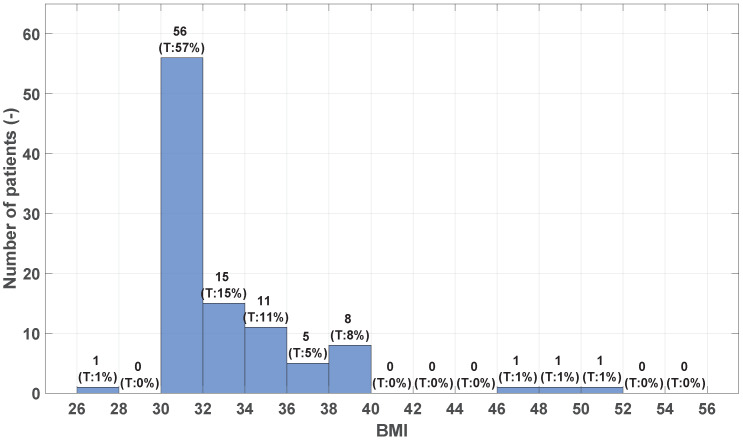
Histogram of BMI.

The surgical incisions in both groups were examined on the 5th, 10th, and 30th days postoperatively. Patients were followed for one year. The primary outcome measure was the occurrence of SSI in both groups, as severe SSI, particularly mesh infection, is a major complication with high morbidity and significant medical, social, and legal consequences [7]. Additionally, we observed factors such as the mean length of hospital stay, hernia area/size, smoking status, BMI (body mass index; for distribution see Figure 2), and sex of the patients, focusing on their influence on SSI rates. The collected data are systematically stored in a structured table, with each row representing an individual patient. The columns correspond to specific variables, which are comprehensively detailed in Table 1. This organization facilitates efficient data management and subsequent analysis.

### 2.2. Statistical Analysis

Given the uniqueness of each patient, we assumed independence across all groups. No patient was operated on twice or treated with both methods. As the normality of the quantitative data was rejected by the Shapiro–Wilk test, indicating that the data were not normally distributed, non-parametric tests were utilized as an alternative to ANOVA. Due to the relatively small sample size and expected low cell counts in the contingency table, we chose the following steps.

For categorical variables, we applied Fisher’s exact test (FET), with the null hypothesis (H0) stating that there is no association between the evaluated phenomenon and the groups being compared. The alternative hypothesis (HA) asserts that there is an association between the evaluated phenomenon and the groups being compared.

For quantitative variables, we tested the null hypothesis (H0) that the medians of all groups are equal. The alternative hypothesis (HA) suggests that the median of at least one group is statistically significantly different. This was evaluated using the Kruskal–Wallis test (KWT). Following the identification of a statistically significant difference among groups by the Kruskal–Wallis test, a post-hoc analysis employing Tukey’s honest significant difference (HSD) criterion was conducted to determine specific group differences.

This statistical approach has been successfully applied in similar studies to assess group differences under conditions of non-normal data distribution and small sample sizes, as demonstrated in [8]. All statistical tests were conducted at a significance level of α = 0.05.

## 3. Results

**Figure 3:**
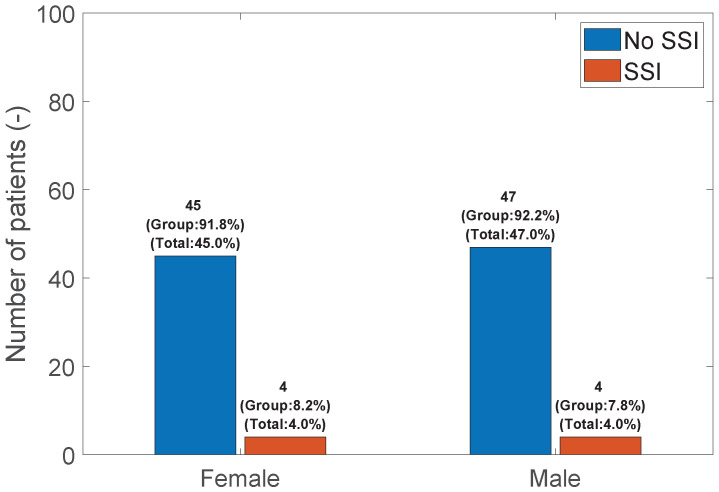
Bar graph of SSI vs. Sex.

Statistical analysis comparing the above parameters between the mentioned groups gave the following results:

SSI vs. Sex: There was no significant relationship between the sex of the patients and the occurrence of SSI (FET; *p*-value = 1.0); see Figure 3.

**Figure 4:**
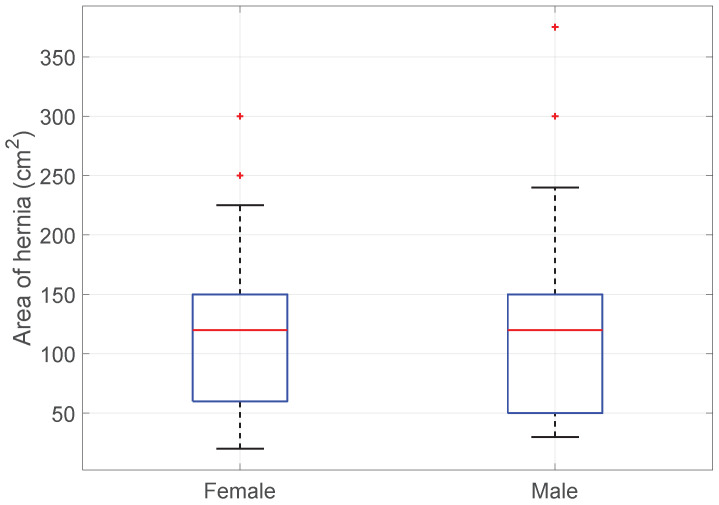
Boxplot of SSI vs. Sex.

SSI vs. Hernia size: Additionally, the size of the hernia did not significantly differ between men and women (KWT; *p*-value > 0.85); see Figure 4.

**Figure 5:**
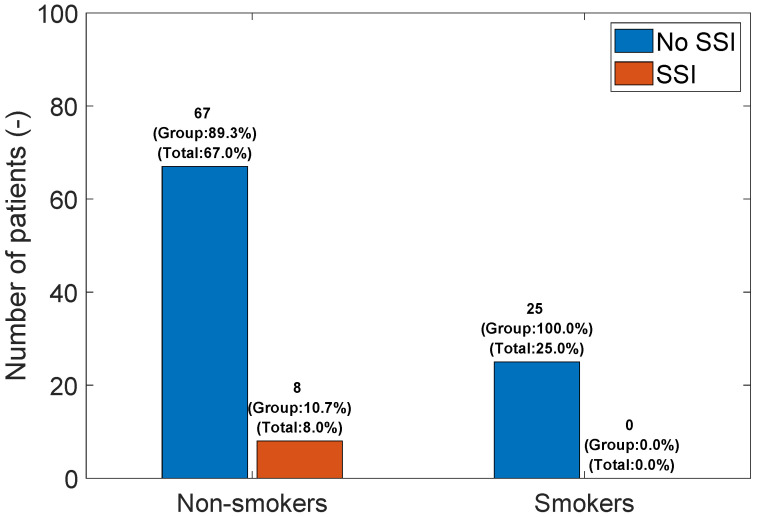
Bar graph of SSI vs. Smoking.

SSI vs. Smoking: Similarly, no statistically significant correlation was found between smoking and SSI (FET; *p*-value = 0.2); see Figure 5.

**Figure 6:**
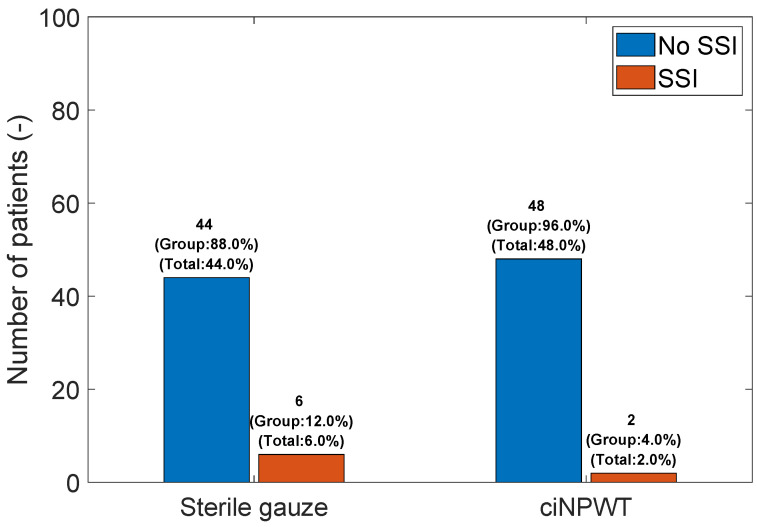
Bar graph of SSI vs. Sterile gauze/ciNPWT.

SSI vs. Sterile gauze/ciNPWT: Surprisingly, the use of ciNPWT did not show a statistically significant difference in the occurrence of SSI compared to the use of sterile gauze (FET; *p*-value = 0.27), see Figure 6.

**Figure 7:**
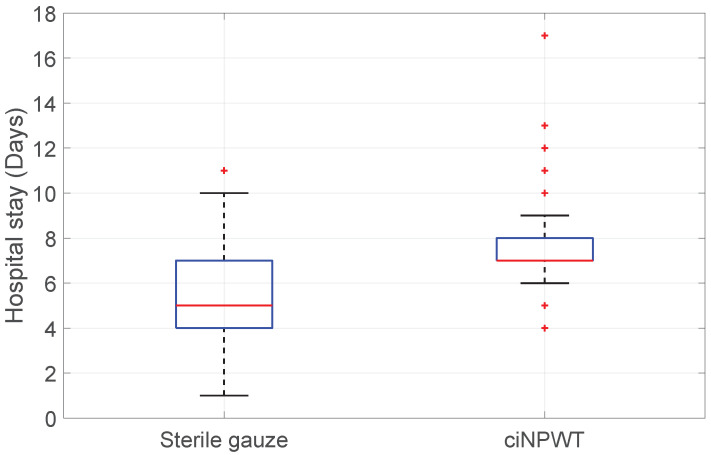
Boxplot of Hospital stay vs. Sterile gauze/ciNPWT.

Hospital stay vs. Sterile gauze/ciNPWT: On the other hand, the application of ciNPWT was associated with a statistically significantly longer hospital stay compared to the use of sterile gauze for treating the surgical incision (KWT; *p*-value < 0.001); see Figure 7.

**Figure 8:**
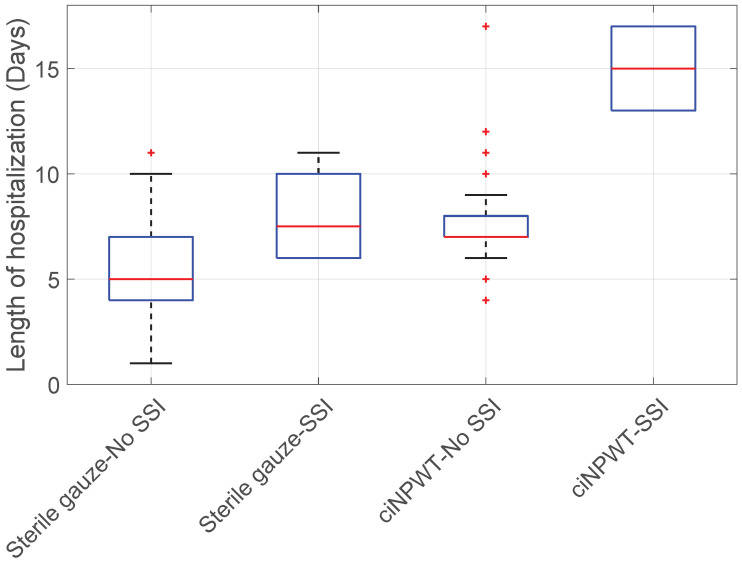
Boxplot of Hospital stay vs. Sterile gauze/ciNPWT vs. SSI.

Hospital stay vs. Sterile gauze/ciNPWT vs. SSI: When comparing the length of hospitalization in patients without SSI across both the sterile gauze and ciNPWT groups, it was found that the mean hospital stay for patients without SSI treated with sterile gauze was significantly shorter than that of patients without SSI treated with ciNPWT (KWT; *p*-value < 0.001), see Figure 8.

Additionally, the hospital stay was significantly longer for patients treated with ciNPWT who developed an SSI compared to those in the sterile gauze group without SSI (KWT + HSD; 0.05 > *p*-value > 0.007), as expected (see Figure 8).

**Figure 9:**
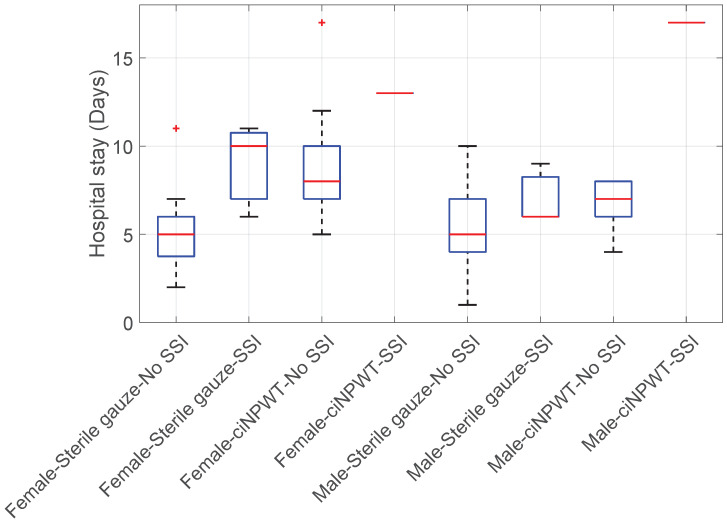
Boxplot of Hospital stay vs. Sterile gauze/ciNPWT vs. SSI vs. Sex.

Hospital stay vs. Sterile gauze/ciNPWT vs. SSI vs. Sex: The analysis revealed that women treated with sterile gauze and without SSI had a significantly shorter length of hospitalization (KWT; *p*-value < 0.001); see Figure 9.

Conversely, women treated with ciNPWT who did not develop SSI had a statistically longer hospital stay compared to men with ciNPWT who also did not develop SSI (KWT + HSD; *p*-value < 0.001, see Figure 9).

**Figure 10:**
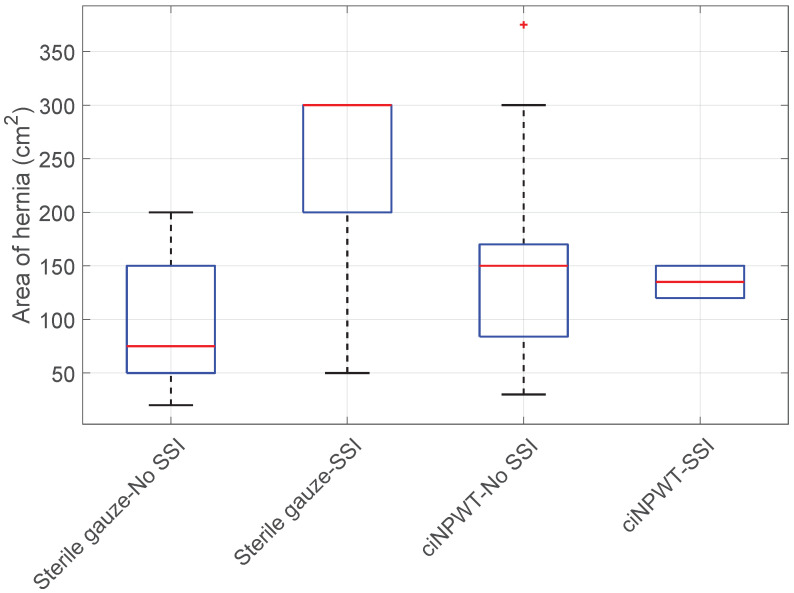
Boxplot of hernia size vs. Sterile gauze/ciNPWT vs. SSI.

Hernia size vs. Sterile gauze/ciNPWT vs. SSI: The Kruskal-Wallis test did reject the null hypothesis. There was a statistically significant difference in hernia size among patients without SSI in both wound care groups. Specifically, the hernia size was significantly larger in patients without SSI treated with ciNPWT compared to those treated with sterile gauze (KWT; *p*-value < 0.001), see Figure 10. Conversely, hernia sizes were significantly larger in patients who developed an SSI and were treated with sterile gauze (KWT + HSD; 0.05 > *p*-value > 0.001); see Figure 10.

**Figure 11:**
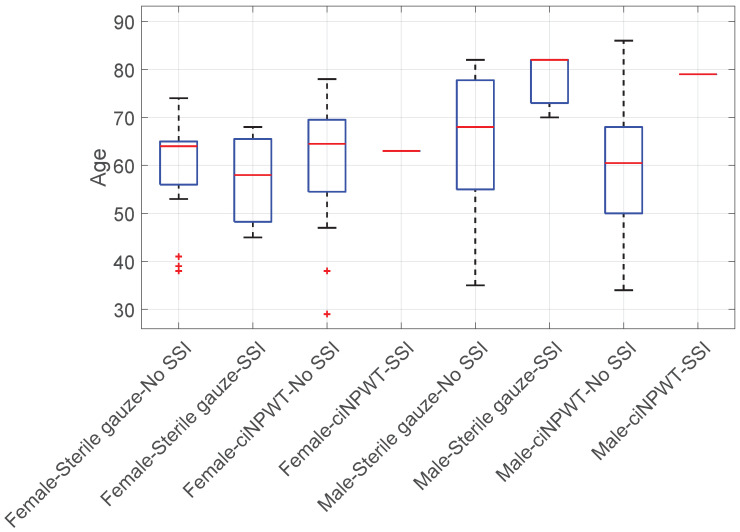
Boxplot of age vs. Sterile gauze/ciNPWT vs. SSI vs. Sex.

Age vs. Sterile gauze/ciNPWT vs. SSI vs. Sex: The Kruskal–Wallis test did not reject the null hypothesis, indicating that age did not significantly influence the occurrence of SSI in either group (KWT; *p*-value > 0.09); see Figure 11.

**Figure 12:**
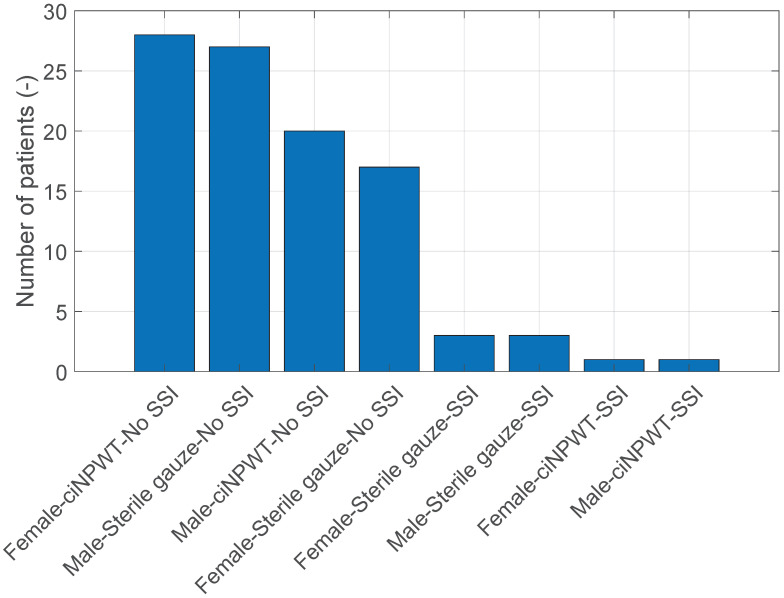
Bar graph of sex vs. Sterile gauze/ciNPWT vs. SSI vs. Number of patients.

For comparison, we present the number of patients in the groups as shown in Figure 12.

**Figure 13:**
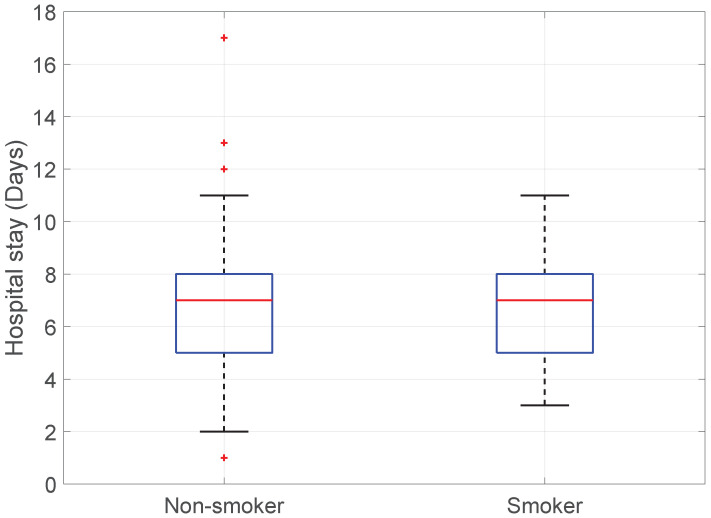
Boxplot of Smoking vs. Hospital stay.

Smoking vs. Hospital stay: Surprisingly, the null hypothesis was not rejected. Smoking status did not significantly affect the length of hospital stay (KWT; *p*-value > 0.99); see Figure 13.

**Figure 14:**
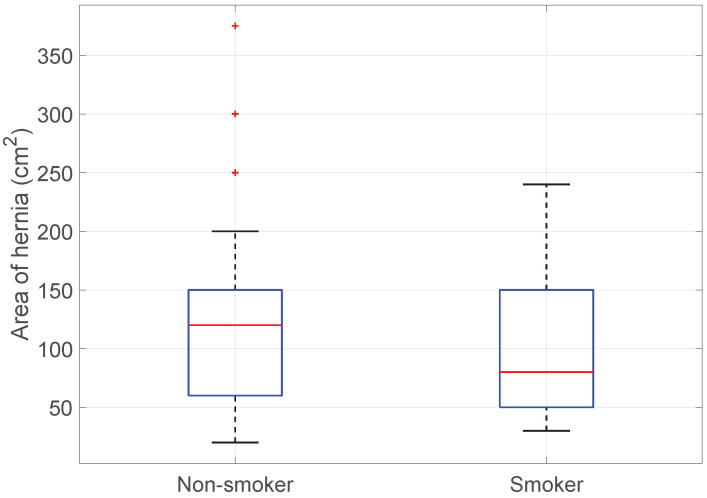
Boxplot of Smoking vs. Hernia size.

Smoking vs. Hernia size: The Kruskal–Wallis test did not reject the null hypothesis and did not show a significant difference in hernia size between smokers and non-smokers. (KWT; *p*-value > 0.34), see Figure 14.

## 4. Discussion

Some of our results were as expected, while others were quite surprising. As far as patient satisfaction and subjective reports are considered, the patients did not complete an evaluation questionnaire regarding patient-reported outcomes. As anticipated, the sex of patients did not play a statistically significant role in the occurrence of SSI, which aligns with the findings of other researchers [9]. Similarly, the age of patients did not significantly influence SSI rates. Although age is a well-known, non-specific factor that can contribute indirectly to postoperative complications through general frailty and comorbidities in elderly patients, its impact on the outcomes of ventral hernia surgery has not been as thoroughly studied as in other surgical procedures. The literature on this topic is limited, with only a few mentions [10]. This may be because very old or severely ill patients are not typically considered for mesh hernia repair. Smoking is another recognized factor associated with surgical complications. However, our study found no significant relationship between smoking and SSI. Interestingly, all eight patients who developed an SSI in our study were non-smokers. This finding contrasts with the existing literature, which generally shows a higher risk of SSI among smokers. This discrepancy is likely due to the relatively small sample size in our study, as larger studies have demonstrated the opposite [11,12]. Additionally, in our study, smokers did not have larger hernias compared to non-smokers, and smoking did not affect the mean length of hospital stay. Regarding the overall occurrence of SSI, 12% (six cases) of patients treated with sterile gauze and only 4% (two cases) of patients treated with ciNPWT developed infectious complications at the surgical site. These results suggest that the use of ciNPWT is associated with a lower infection rate. Unfortunately, this difference was not statistically significant, likely due to the limited size of our study. However, meta-analyses by Berner-Hansen et al. [2] and Guo and Cheng [13] report a statistically significant reduction in SSI with the use of protective NPWT after hernia surgery. On the other hand, the use of protective NPWT in postoperative wound care was associated with a statistically significant increase in hospital stay compared to patients whose surgical incisions were treated with sterile gauze. The average length of hospital stay was 5.6 days for patients treated with sterile gauze, but it increased to 8.2 days for those who received ciNPWT. Interestingly, among patients without SSI treated with sterile gauze, women spent significantly less time in the hospital than men (mean: F-5.1 vs. M-5.4 days). This trend was reversed in the ciNPWT group, where women without SSI had a statistically longer hospital stay than men (mean: F-8.6 vs. M-6.9 days). Notably, the size of the hernia was not significantly different between women and men, which suggests that other factors may contribute to this difference. The longer hospital stay in the ciNPWT group could be only partly explained by the nature of the therapy itself, which required a uniform 5-day inpatient period. This difference in length of stay persisted even when focusing on patients with SSI. Those with SSI treated with sterile gauze had a shorter hospital stay (mean of 8 days), while those treated with ciNPWT had a significantly longer stay (mean of 15 days). However, many of these patients were clinically stable and could have been discharged earlier. The generally longer hospital stay in the ciNPWT group was thoroughly analyzed, but no specific factors directly responsible for such a long stay could be identified. The conclusion was that the longer stay was mainly caused by the safety concerns of the personnel “not seeing the wound” for long enough before dismissal, with little actual medical rationale supporting a prolonged stay. A key practical advantage of ciNPWT was demonstrated in relation to hernia size and SSI. The hernia size in patients without SSI was statistically larger in the ciNPWT group compared to those treated with sterile gauze. Conversely, in patients with SSI, those treated with sterile gauze had significantly larger hernias than those treated with ciNPWT. This finding suggests that ciNPWT may make hernia surgery safer as hernia size increases, which has direct implications for patient management and surgical indications. In other words: the larger the hernia, the more advantageous ciNPWT becomes. This is arguably the most important result of our study.

## 5. Conclusions

The strength of this study is limited by the relatively small sample size. A larger-scale study would likely have produced more statistically significant results. Nevertheless, our findings are largely consistent with the existing literature. The application of ciNPWT appears to be a safe and effective method for surgical wound management in patients undergoing elective procedures. However, further research is needed to determine the optimal duration of ciNPWT, as the 5-day application period used in this study may be unnecessarily long from both financial and medical perspectives. A follow-up study focused on the optimal ciNPWT duration, together with a patient satisfaction questionnaire, is currently being designed. Importantly, our study demonstrated a positive effect of ciNPWT on ventral hernia surgery, particularly in terms of reducing SSI rates. This positive effect becomes increasingly significant with larger hernia sizes, a finding that is relatively unique in the literature.

## Figures and Tables

**Table 1:** Table of patient data variables.

Column of Table	Description
Patient ID	Generated unique anonymous number
Age	Years
Sex	Female/Male
ASA Classification	1–6 (in this case: 2–3)
Duration of Surgery	Minutes
Hernia Area	cm^2^
Body Mass Index (BMI)	kg/m^2^
Diabetes Mellitus (DM)	No/Yes
Smoker	No/Yes
Hospital stay	Days
Chronic Obstructive Pulmonary Disease (CHOPN)	No/Yes
Method	Sterile gauze/ciNPWT
Surgical site infections (SSIs)	No/Yes; Yes, if there is an infection at some checkup
Number of Days with NPWT at 90 mmHg	Days (in this case, always 5 days of treatment.)

## Data Availability

Anonymized data used for this article will be provided by the authors upon request.

## Abbreviations

The following abbreviations are used in this manuscript:

ANOVA	Analysis of variance
ASA	assessment of the general condition of the patient before surgery according to the American Society of Anesthesiology
BMI	body mass index
ciNPWT	Closed-incision negative-pressure wound therapy
CHOPN	chronic obstructive pulmonary disease
FET	Fisher’s exact test
HSD	honest significant difference
KWT	Kruskal–Wallis test
NPWT	negative pressure therapy
ORIF	open reduction, internal fixation
SSI	surgical site infection
V.A.C.	vacuum wound closure
VEGF	vascular endothelial growth factor
